# Transforming joint replacement with open robotics: A call for change!

**DOI:** 10.1051/sicotj/2025059

**Published:** 2025-12-10

**Authors:** Vaibhav Bagaria, Raju Vaishya

**Affiliations:** 1 Director and Consultant, Department of Orthopedic Surgery, Sir H N Reliance Foundation Hospital, Girgaum Mumbai Maharashtra India; 2 Senior Consultant Orthopaedics, Indraprastha Apollo Hospitals Sarita Vihar New Delhi 110076 India

**Keywords:** Open source Robotics, RATKA, Robotic Hip replacement, Robotic UKR, Innovations

As robotic-assisted technology continues to refine joint replacement surgeries through its precision and potential for enhanced implant longevity, a critical question emerges: Are monopolistic practices stifling medical innovation for commercial gain? This ongoing discussion seeks to answer this question, challenge the existing framework, and explore the potential of a new paradigm.

## Questioning the current system

1. *Surgeon autonomy vs. corporate agreements*: One of the primary concerns surrounding robotic systems in joint replacement is the limitation placed on skilled surgeons due to corporate agreements with specific implant manufacturers. Many current systems bind surgeons to particular implants, thereby restricting their autonomy. This raises an essential question: Are these limitations genuinely in the best interest of patient care, or do corporate profits primarily drive them? This issue is critical as it undermines the surgeon’s ability to select the best patient options based on anatomical and clinical needs [1–3].
2. *Innovation at the cost of monopoly*: A prevalent concern is whether true innovation can flourish under the control of a few dominant companies. Proprietary systems often hinder technological evolution, preventing advancements that could benefit a broader range of patients. For instance, it has been documented that in highly monopolised markets, the lack of competition can stifle creativity and lead to homogenised approaches to medical treatment. This raises the question of how we can encourage a more vibrant ecosystem of technological advancement that benefits all stakeholders [4, 5].
3. *Economic barriers to advanced care*: The high cost of proprietary robotic systems raises significant questions about healthcare equity. Many institutions and patients cannot access advanced robotic technologies due to their prohibitive costs, leading to disparities in care. Economic barriers suggest that current business models are more protective of the status quo, favouring a few entities rather than promoting widespread access to innovative healthcare solutions [6–8].
4. *Global and national disparities in access*: While technological advancements such as robotic-assisted joint replacement surgery promise improved outcomes, their adoption reveals striking disparities both within nations and across the globe. Countries like the United States and India, with their large private healthcare sectors, have rapidly embraced robotic systems [9]. In contrast, nations with predominantly socialist or centralized healthcare models, such as the UK or Canada, have adopted these technologies more conservatively, constrained by budgetary allocations and cost-effectiveness thresholds. Even within a single country, the distribution of robotic platforms is skewed. Larger corporate hospitals in metropolitan regions are far more likely to offer robotic options, whereas public and rural institutions often lag. This uneven landscape underscores the urgent need for affordable, accessible, and flexible solutions like open robotic platforms, which could bridge these gaps and democratize access to advanced care [5].

## Learning from other industries

The success of open platforms in sectors like information technology serves as a compelling case for their application in healthcare. The open-source model employed in the tech industry, epitomised by platforms like Android, allows for competition and innovation, leading to diverse products and lower costs [7]. The automotive industry’s Open Automotive Alliance also illustrates how a common platform can encourage innovation and ensure safety [3]. Such examples indicate that an open platform model could similarly stimulate growth and technological advancement within healthcare [6].

## Acknowledging the counterarguments

Despite the advantages of an open platform, there are valid counterarguments. Proponents of the proprietary model argue that the high costs associated with research and development for advanced robotic systems are justified. They contend that these expenses are often recouped through the sales of proprietary implants, and without financial incentives from exclusive deals, companies may lack the capital necessary for future innovations. This perspective warns that a sudden shift to an open platform could disrupt the economy and the stability currently supporting ongoing technological advancements [2, 10].

## Evidence from recent literature

Bagaria and Poduval (2023) emphasised the importance of open-source approaches in robotic joint replacement, proposing a universal system for implant sizing and nomenclature [4]. They argue that standardisation would make robotic technology more accessible and affordable, supporting the editorial’s call for open platforms. Zhuang et al. (2023) compared open and closed platform robotic systems in total hip arthroplasty (THA) and found no significant differences in surgical time, blood loss, or clinical outcomes (e.g., Harris Hip Score). Both systems demonstrated similar learning curves and safety profiles, suggesting that open platforms can match the performance of proprietary systems while offering greater flexibility [11]. Yang and Seon (2023) reviewed the landscape of surgical robotics in orthopaedics, noting the existence of both closed- and open-platform systems. Regardless of platform, they stress that the main goal is to improve surgical accuracy and outcomes, but highlight that the surgeons must weigh the benefits and drawbacks of each system to meet patient needs [12].

## Proposing a balanced solution

Comprehensive Open Robotics Ecosystem (CORE): Considering these factors, the Comprehensive Open Robotics Ecosystem (CORE) model should strive to balance the perspectives of all stakeholders: Surgeons, hospitals, implant companies, start-ups, and most importantly, patients [13, 14]. This model aims to cultivate an environment where innovation, surgeon autonomy, and patient-specific care are paramount, while also recognising the financial realities manufacturers face. Introducing open platform principles could allow existing companies to adapt economically while fostering innovations and collaborations, enhancing overall patient care.

A summary of the pros and cons of open vs. closed platform systems is presented in Table 1. A proof-of-concept open-source platform for cooperative, semi-autonomous robotic surgery will demonstrate feasibility and accuracy in preclinical testing, suggesting that open-source approaches can be viable for various surgical applications.

**Table 1:** Pros and cons of open vs. closed platform systems.

Aspect	Open platform systems	Closed platform systems
Surgeon choice	Broad, implant-agnostic	Limited to specific implants
Innovation	Encourages, via collaboration	Potentially restricted
Cost/access	Potentially lower, broader	Higher, limited access
Clinical outcomes	Comparable to closed systems	Proven, but not superior
Learning curve	Similar to closed systems	Similar

## Conclusion

As we engage in this crucial debate regarding the future of robotic surgery, it is imperative to weigh the benefits of open innovation against the practical economic concerns of industry stakeholders. The CORE model serves as both a call to action and a framework for dialogue, advocating for a gradual transition that safeguards patient interests while allowing the industry to innovate effectively. Leading this discussion with open-mindedness can help identify a path that aligns technological advancement with the highest standards of patient care and ethical practice.

## Data Availability

Data availability is not applicable.
